# Supplementary material for: Integration of phylogenomics and molecular modeling reveals lineage-specific diversification of toxins in scorpions
Source: PeerJ. 2018 Nov 14;6:e5902. doi: 10.7717/peerj.5902 (PMC6240337; doi:10.7717/peerj.5902)
Supplement: Supplemental Information 2 [file peerj-06-5902-s002.pdf]

Supplementary Files

Integration of phylogenomics and molecular modeling reveals lineage-specific diversification of toxins in scorpions

Carlos E. Santibáñez López,[*1,2] Ricardo Kriebel,[3] Jesús A. Ballesteros[1], Nathaniel Rush,[4] Zachary Witter,[4] John Williams,[4] Daniel Janies[4] and Prashant P. Sharma[1]

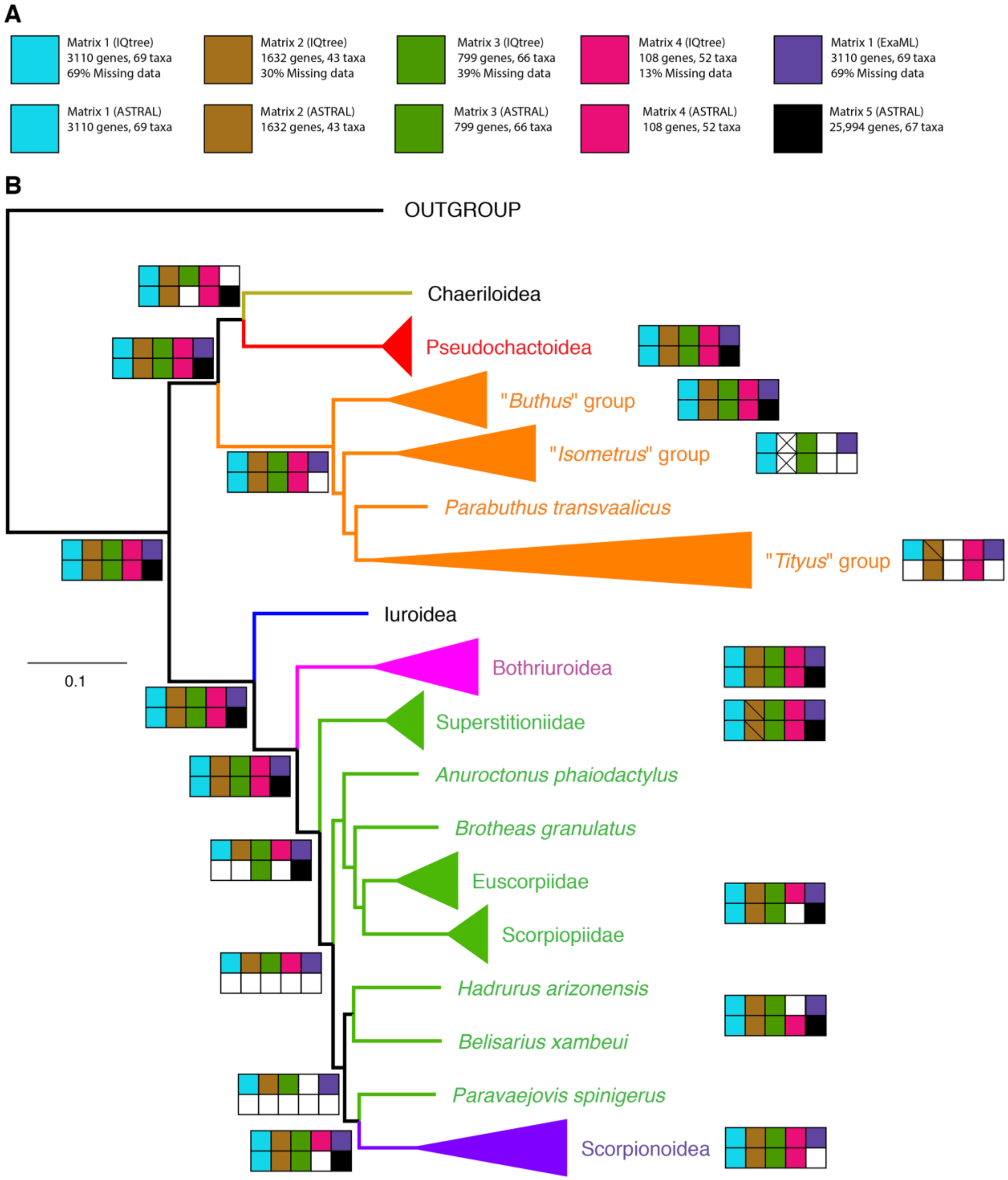

Figure S1. Phylogenomic analyses of Scorpiones. (a) Supermatrix composition, including number of genes and taxa. (b) Tree topology of Scorpiones inferred from ML (IQ-TREE) analysis of Matrix 1. Navajo plots indicate recovery of a given node in the corresponding analysis. Cross squared indicates taxa from that clade was not included in the analyses. \ indicates the clade was represented only by one terminal.

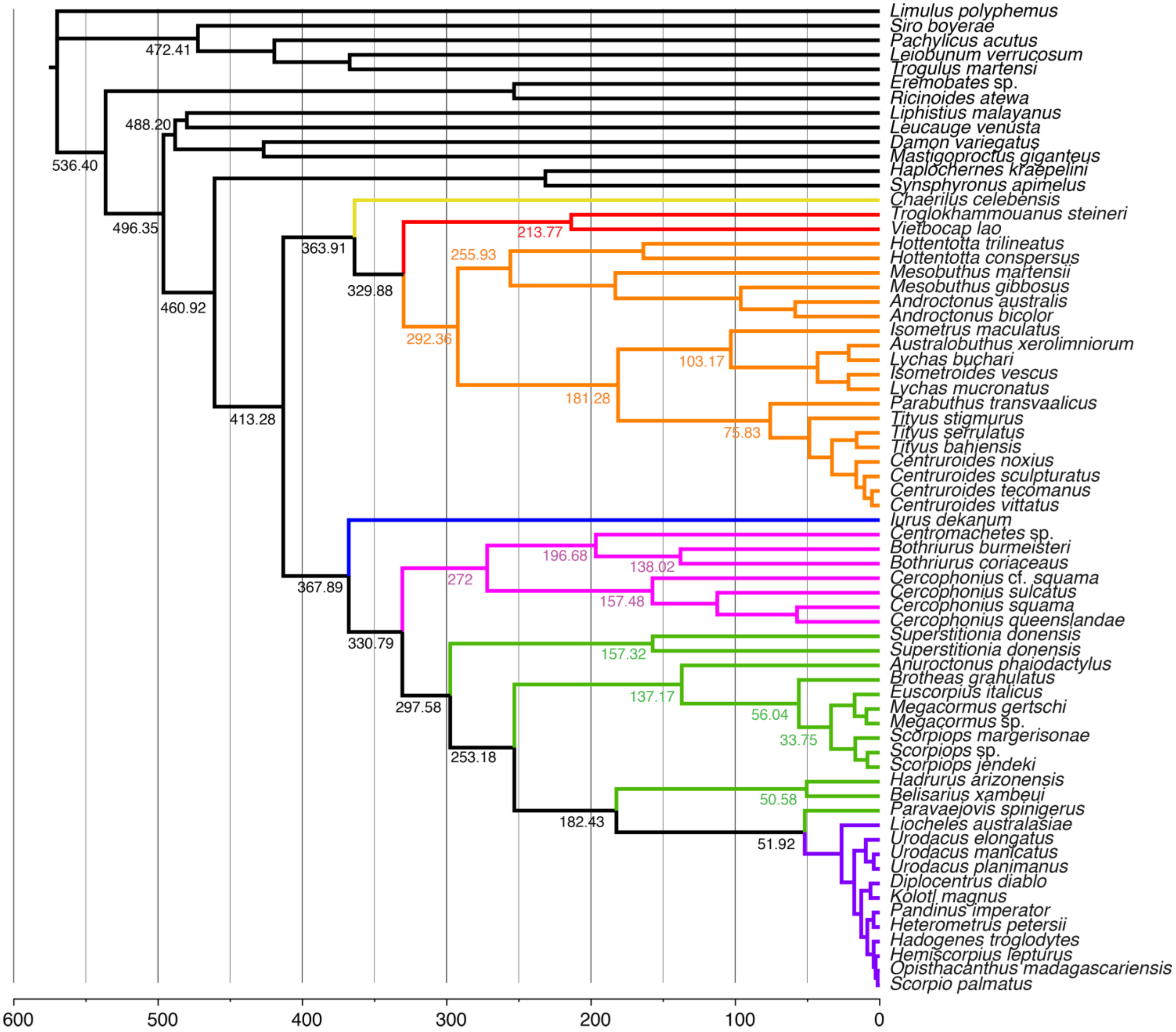

Figure S2. Evolutionary tree with times of divergence, based on the maximum likelihood tree topology inferred using Matrix 1. Calibrated using a penalized likelihood function implemented in the *chronos* function of the R package *ape* under a relaxed model and lambda of 0.5.

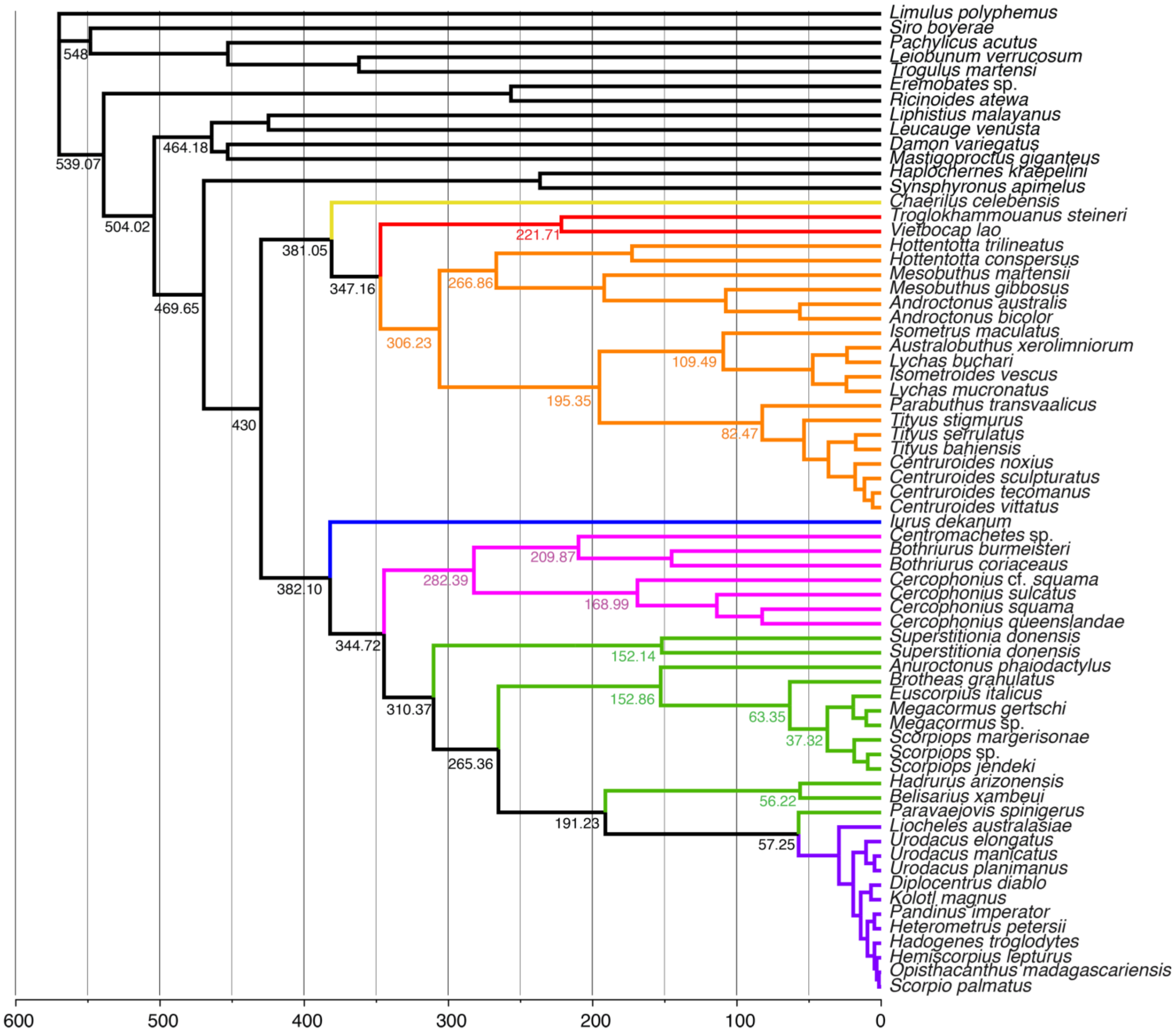

Figure S3. Evolutionary tree with times of divergence, based on the maximum likelihood tree topology inferred using Matrix 1. Calibrated using a penalized likelihood function implemented in the *chronos* function of the R package *ape* under a relaxed model and lambda of 1.0.

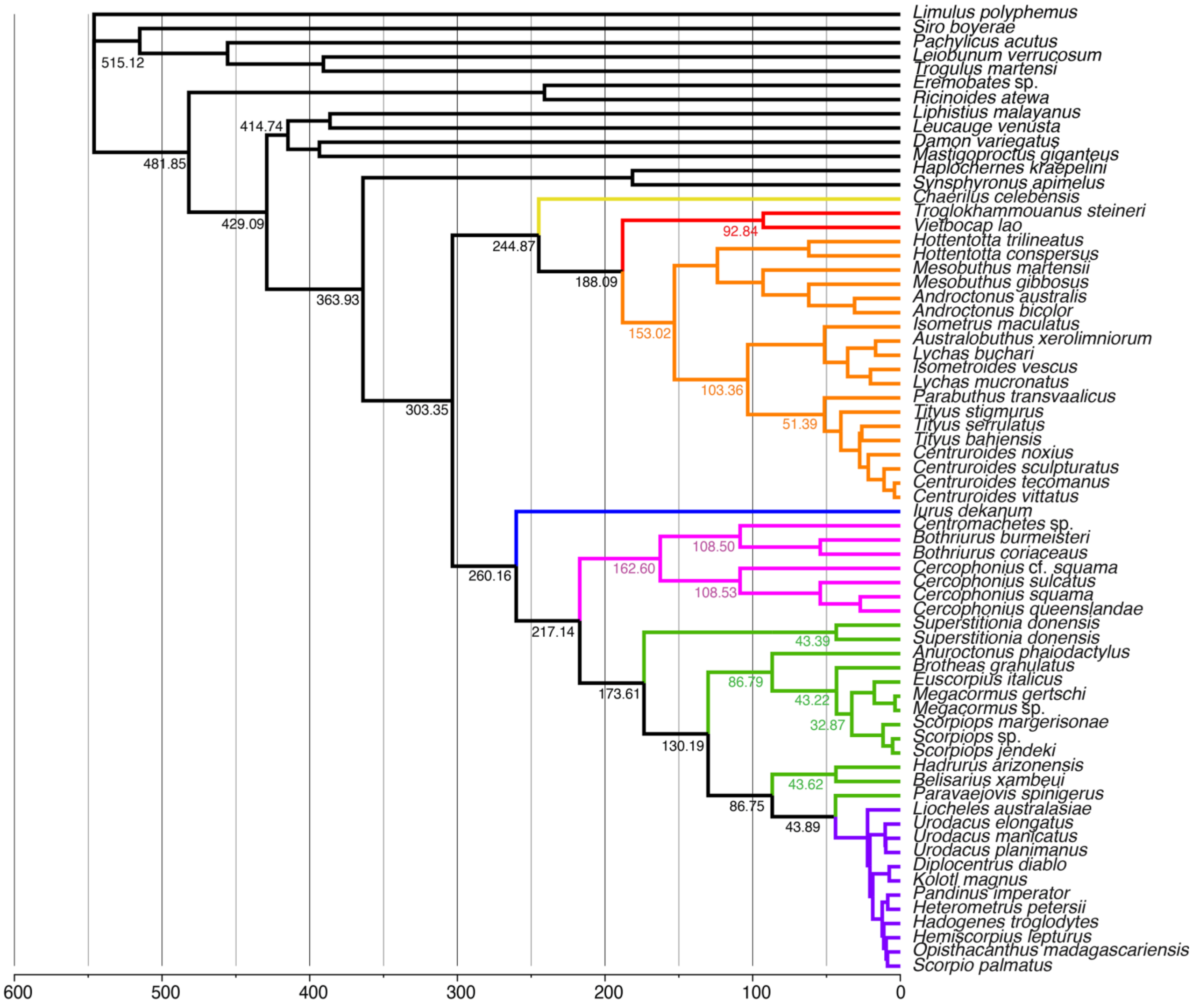

Figure S4. Evolutionary tree with times of divergence, based on the maximum likelihood tree topology inferred using Matrix 1. Calibrated using a penalized likelihood function implemented in the *chronos* function of the R package *ape* under a correlated model and lambda of 0.5.

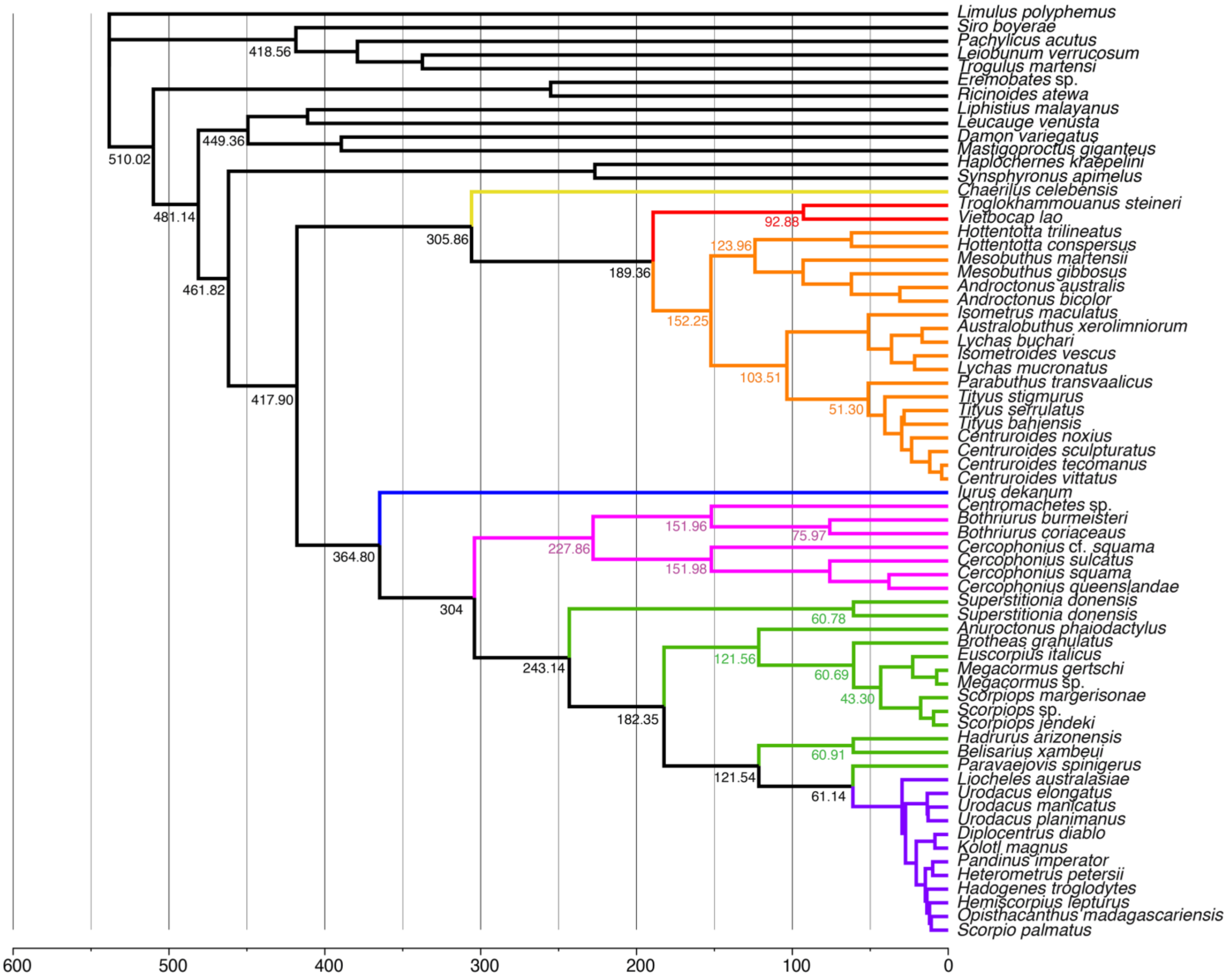

Figure S5. Evolutionary tree with times of divergence, based on the maximum likelihood tree topology inferred using Matrix 1. Calibrated using a penalized likelihood function implemented in the *chronos* function of the R package *ape* under a correlated model and lambda of 1.0.

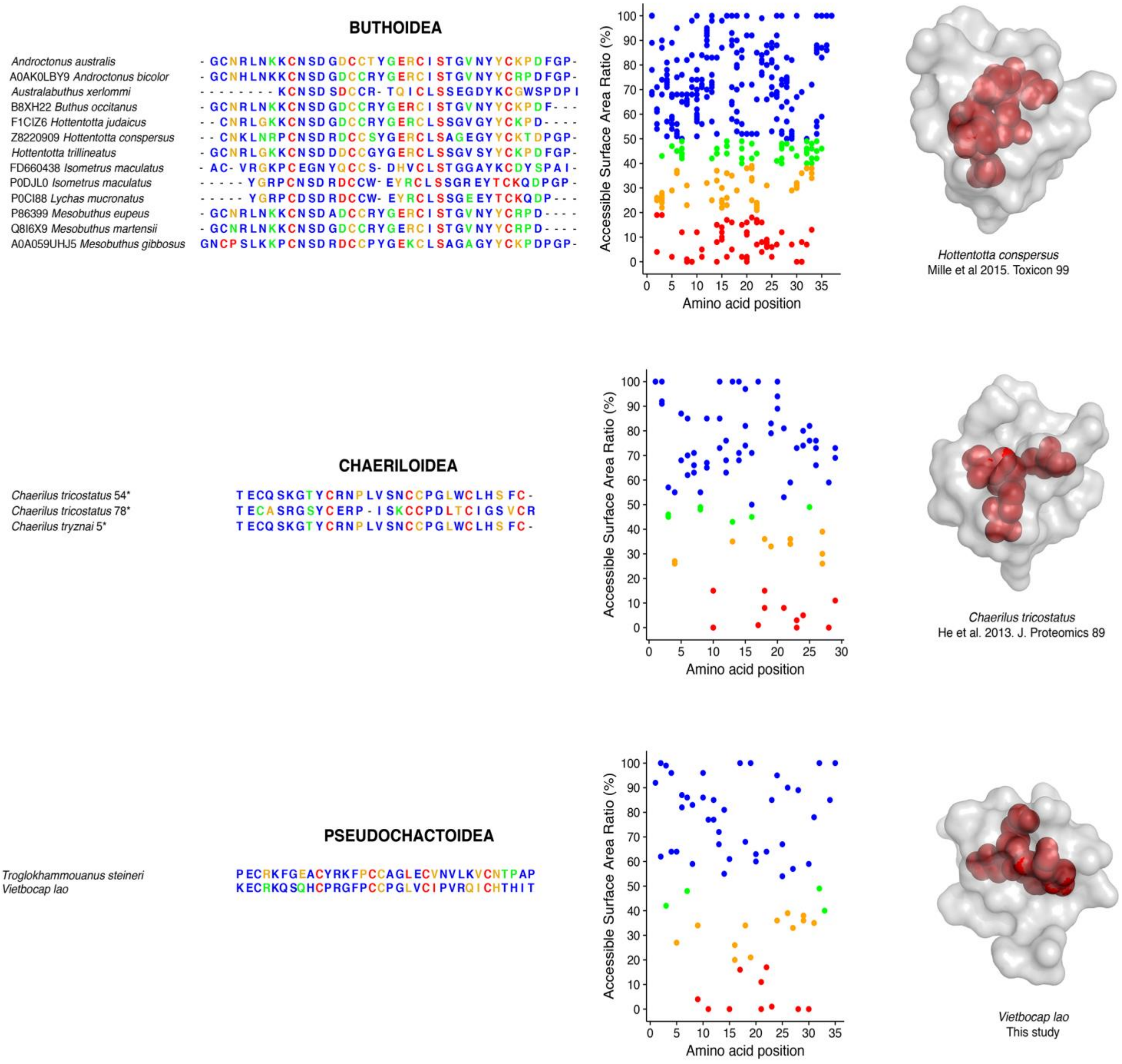

Figure S6. LKTx peptide sequences. Left: Multiple sequence alignment by scorpion superfamily. Center: Plot of amino acid positions (x-axis) and the accessible surface ratio (ASR) ratio (y-axis) indicating the exposure level of each amino acid by colors. Categories: exposed (>50%, blue), potentially exposed (>40%, green), potentially buried (20%> x <40%, orange), buried (<20%, red). Right: Three-dimensional model of an LTKx peptide exemplar with the ASR (in gray) and the buried core amino acids (in red).

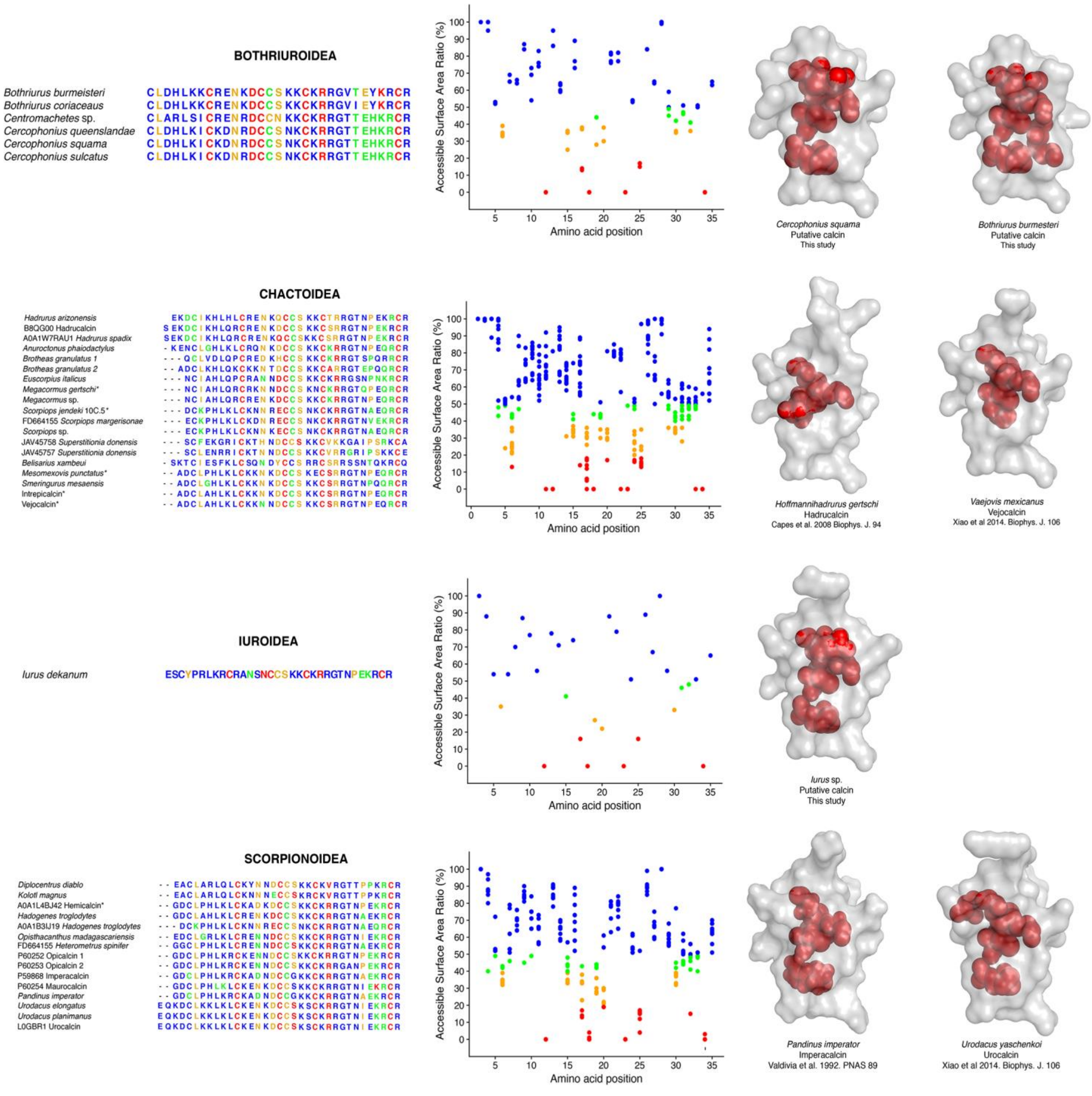

Figure S7. Calcin peptide sequences. Left: Multiple sequence alignment by scorpion superfamily. Center: Plot of amino acid positions (x-axis) and the accessible surface ratio (ASR) ratio (y-axis) indicating the exposure level of each amino acid by colors. Categories as in Figure S6. Right: Three-dimensional model of calcin exemplar with the ASR (in gray) and the buried core amino acids (in red).

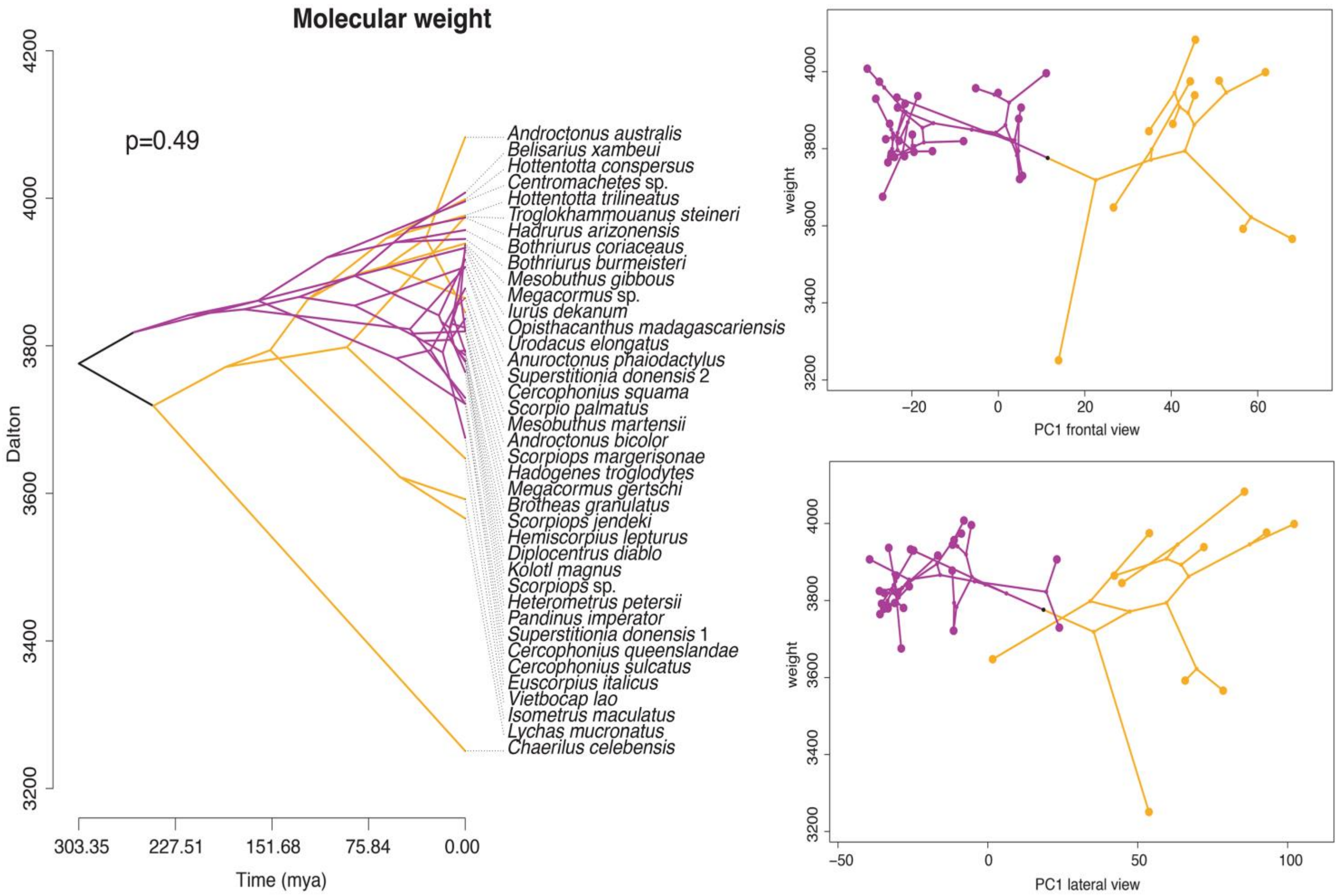

Figure S8. Morphometric analyses of the 3D structure of calcins (purple) and LKTx (orange) in scorpion venom. (*a*) Visualization of the molecular weight data as a function of phylogenetic relationships recovered from the dated molecular tree; horizontal axis indicates the time of divergence and vertical axis indicates the molecular weight in Daltons. (b-c) Visualization of phylogenomic tree on the morphospace of the frontal (b) and lateral (c) views, and the molecular weight showing the distinction between LKTx and calcins; horizontal axis indicates PC1 values and vertical axis indicate the molecular weight in Daltons.

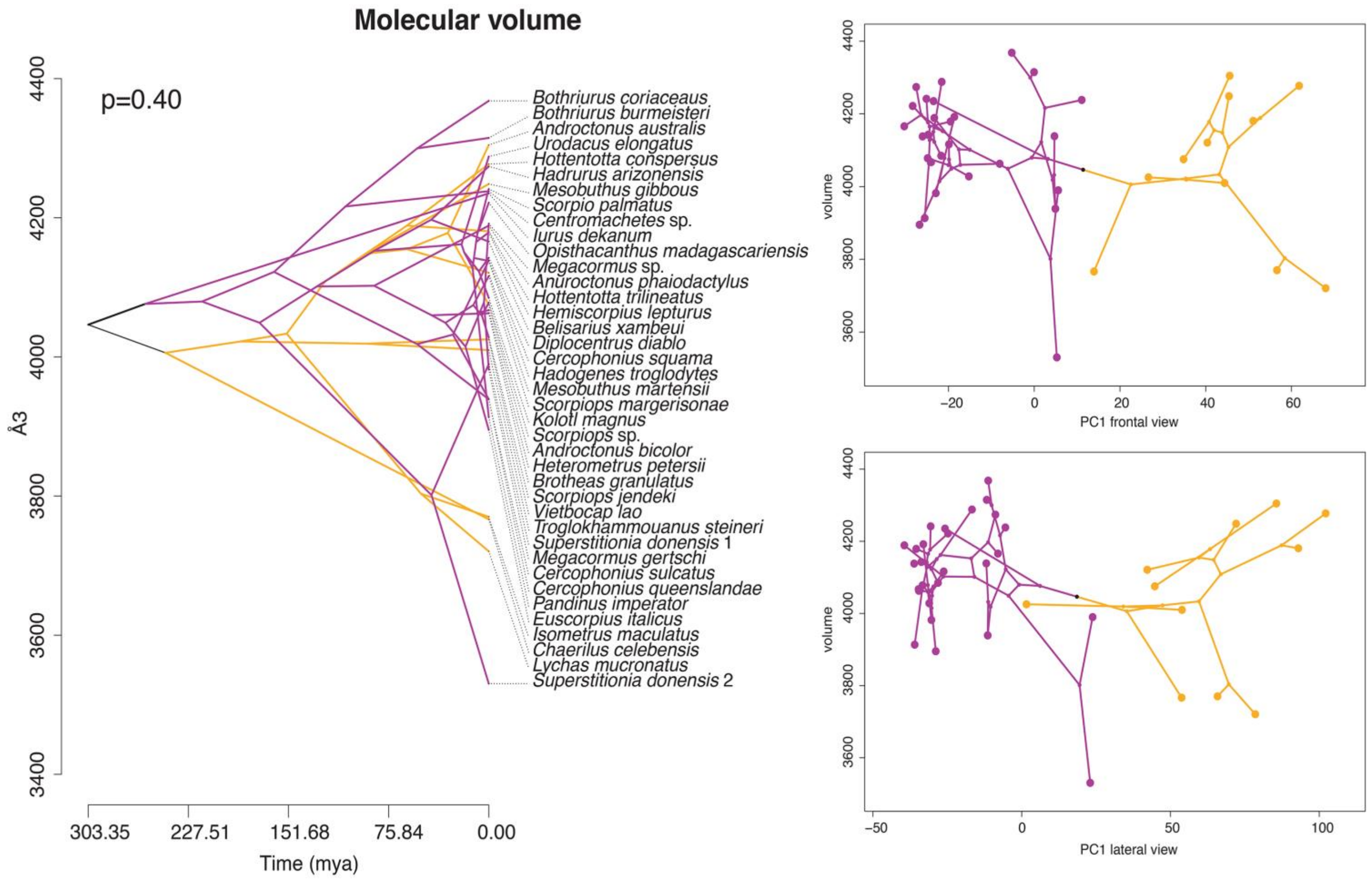

Figure S9. Morphometric analyses of the 3D structure of calcins (purple) and LKTx (orange) in scorpion venom. (*a*) Visualization of the molecular volume data as a function of phylogenetic relationships recovered from the dated molecular tree; horizontal axis indicates the time of divergence and vertical axis indicates the volume in cubic angstrom. (b-c) Visualization of phylogenomic tree on the morphospace of the frontal (b) and lateral (c) views, and the molecular volume showing the distinction between LKTx and calcins; horizontal axis indicates PC1 values and vertical axis indicate the molecular volume in cubic angstrom.

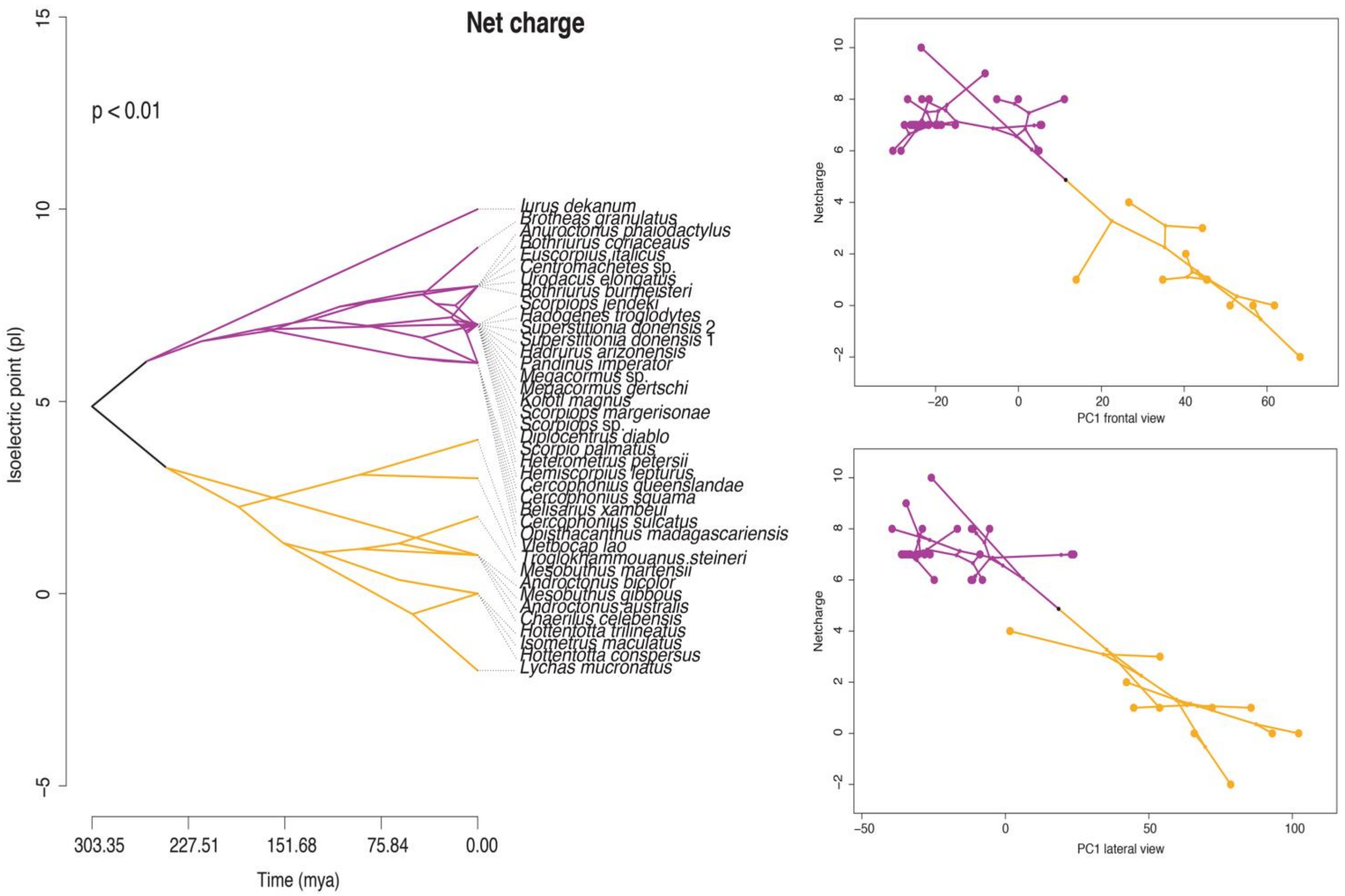

Figure S10. Morphometric analyses of the 3D structure of calcins (purple) and LKTx (orange) in scorpion venom. (*a*) Visualization of the molecular weight data as a function of phylogenetic relationships recovered from the dated molecular tree; horizontal axis indicates the time of divergence and vertical axis indicates the isoelectric point. (b-c) Visualization of phylogenomic tree on the morphospace of the frontal (b) and lateral (c) views, and the molecular weight showing the distinction between LKTx and calcins; horizontal axis indicates PC1 values and vertical axis indicate the isoelectric point.

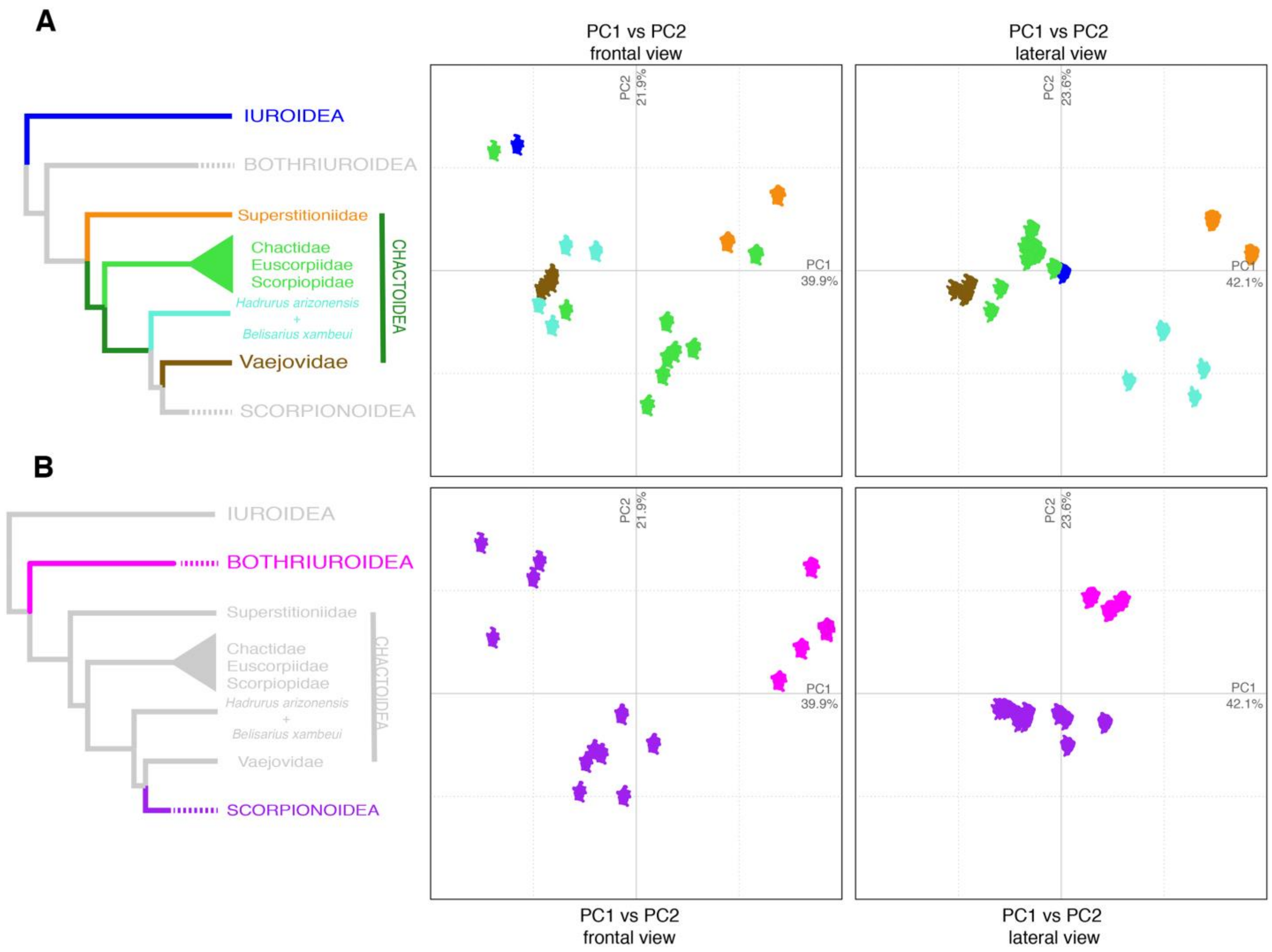

Figure S11. Morphometric analyses of the 3D structure of calcins. (*a*) Left: ML phylogenomic tree showing Iurida superfamilies (colors); Right: Visualization of the morphospaces showing the variation between calcins of Iuroidea and Chactoidea superfamilies, in principal components of frontal (upper row, center) and lateral (upper row, right) views. (*b*) Corresponding analysis comparing the 3D structure of calcins in Scorpionoidea and Bothriuroidea in frontal (lower row, center) and lateral (lower row, right) views.

Supplementary References

Abdel-Rahman, M.A., Quintero-Hernández, V. and Possani, L.D. 2013. Venom proteomic and venomous glands transcriptomic analysis of the Egyptian scorpion *Scorpio maurus palmatus* (Arachnida: Scorpionidae). *Toxicon* 74:193–207.

Almeida, D.D., Scortecci, K.C., Kobashi, L.S., Agnez-Lima, L.F., Medeiros, S.S, Silva-Junior, A.A., Junqueira-de-Azevedo Ide, L. and Fernandes-Pedrosa M de F. 2012. Profiling the resting venom gland of the scorpion *Tityus stigmurus* through a transcriptomic survey. BMC Genomics 13:362.

Alvarenga, E.R., Mendes, T.M., Magalhaes, B.F., Siqueira, F.F., Dantas, A.E., Barroca, T.M., Horta, C.C. and Kalapothakis, E. 2012. Transcriptome analysis of the *Tityus serrulatus* scorpion venom gland. *Open Journal of Genetics* 2:210-220.

Cao, Z.J., Xie, Y., Dai, C., Zhu, S.Y., Yin, S.J., Wu, Y.L. and Li, W.X. 2006. Cloning and characterization of a novel calcium channel toxin-like gene BmCa1 from Chinese scorpion *Mesobuthus martensii* Karsch. *Peptides* 27:1235-1240.

Cao, Z., Yu, Y., Wu, Y., Hao, P., Di, Z., He, Y., Chen, Z., Yang, W., Shen Z., He X. et al. 2013. The genome of *Mesobuthus martensii* reveals a unique adaptation model of arthropods. *Nature Communications* 4:2602.

Chen, Z., Hu, Y., Han, S., Yin, S., He, Y., Wu, Y., Cao, Z. and Li, W. 2011. ImKTx1, a new Kv1.3 channel blocker with a unique primary structure. *Journal of Biochemical and Molecular Toxicology* 25:244-251.

de Oliveira, U.C., Candido, D.M., Dorce, V.A. and Junqueira-de-Azevedo Ide, L. 2015. The transcriptome recipe for the venom cocktail of *Tityus bahiensis* scorpion. *Toxicon* 95:52-61.

Diego-García, E., Caliskan, F. and Tytgat, J. 2014. The Mediterranean scorpion *Mesobuthus gibbosus* (Scorpiones: Buthidae): transcriptome analysis and organization of the genome encoding chlorotoxin-like peptides. *BMC Genomics* 15:295.

Fajloun, Z., Kharrat, R., Chen, L., Lecomte, C., Di Luccio, E., Bichet, D., El Ayeb, M., Rochat, H., Allen, P.D., Pessah, I.N., De Waard, M. and Sabatier, J.M. 2000. Chemical synthesis and characterization of maurocalcine, a scorpion toxin that activates Ca(2+) release channel/ryanodine receptors. *FEBS Letters* 269:179-185.

He, Y., Zhao, R., Di, Z., Li, Z., Xu, X., Hong, W., Wu, Y., Zhao, H., Li, W. and Cao, Z. 2013 Molecular diversity of Chaerilidae venom peptides reveals the dynamic evolution of scorpion venom components from Buthidae to non-Buthidae. *Journal of Proteomics* 89:1-14.

Hedin, M., Starrett, J., Akhter, S., Schonhofer, A.L., Shultz, J.W. 2012. Phylogenomic resolution of Paleozoic divergences in harvestmen (Arachnida: Opiliones) via analysis of next-generation transcriptome data. *PLoS ONE* 7:e42888.

Kazemi-Lomedasht F, Khalaj V, Bagheri KP, Behdani M, Shahbazzadeh D. 2017. The first report on transcriptome analysis of the venom gland of Iranian scorpion *Hemiscorpius lepturus*. *Toxicon* 125:123-130.

Luna-Ramírez, K., Quintero-Hernández, V., Vargas-Jaimes, L., Batista, C.V., Winkel, K.D. and Possani, L.D. 2013. Characterization of the venom from the Australian scorpion *Urodacus yaschenkoi*: molecular mass analysis of components, cDNA sequences and peptides with antimicrobial activity. *Toxicon* 63:44-54.

Ma, Y., Zhao, R., He, Y., Li, S., Wu, Y., Cao, Z. and Li, W. 2009. Transcriptome analysis of the venom gland of the scorpion *Scorpiops jendeki*: implication for the evolution of the scorpion venom arsenal. *BMC Genomics* 10:290–15.

Ma, Y., Zhao, Y., Zhao, R., Zhang, W., He, Y., Wu, Y., Cao, Z., Guo, L. and Li, W. 2010. Molecular diversity of toxic components from the scorpion *Heterometrus petersii* venom revealed by proteomic and transcriptome analysis. *Proteomics* 10:2471-2485.

Ma, Y., He, Y., Zhao, R., Wu, Y., Li, W. and Cao, Z. 2012. Extreme diversity of scorpion venom peptides and proteins revealed by transcriptomic analysis: Implication for proteome evolution of scorpion venom arsenal. *Journal of Proteomics* 75:1563–76.

Mille, B.G., Peigneur, S., Predel, R. and Tytgat, J. 2015. Transcriptomic approach reveals the molecular diversity of *Hottentotta conspersus* (Buthidae) venom. *Toxicon* 99:73-79.

Morgestern, D., Rohde, B.H., King, G.F., Tal, T., Sher, D. and Zlotkin, E. 2011. The tale of a resting gland: transcriptome of a replete venom gland from the scorpion *Hottentotta judaicus*. *Toxicon* 57:695-703.

Ono, S., Kimura, T. and Kubo, T. 2011. Characterization of voltage-dependent calcium channel blocking peptides from the venom of the tarantula *Grammostola rosea*. *Toxicon* 58:265-276.

Quintero-Hernández, V., Ramírez-Carreto, S., Romero-Gutiérrez, M.T., Valdez-Velázquez, L.L., Becerril, B., Possani, L.D. and Ortiz, E. 2013. Transcriptome analysis of scorpion species belonging to the *Vaejovis* genus. *PLoS ONE* 10:e0117188.

Rendon-Anaya, M., Delaye, L., Possani, L.D. & Herrera-Estrella, A. 2012. Global transcriptome analysis of the scorpion *Centruroides noxius*: New toxin families and evolutionary insights from an ancestral scorpion species. *PLoS ONE* 7:e43331.

Rokyta, D.R. and Ward, M.J. 2017. Venom-gland transcriptomics and venom proteomics of the black-back scorpion (*Hadrurus spadix*) reveal detectability challenges and an unexplored realm of animal toxin diversity. *Toxicon* 128:23-37.

Ruiming, Z., Yibao, M., Yawen, H., Zhiyong, D., Yingliang, W., Zhijian, C., Wenxin, L. 2010. Comparative venom gland transcriptome analysis of the scorpion *Lychas mucronatus* reveals intraspecific toxin gene diversity and new venomous components. *BMC Genomics* 11:452.

Schwartz, E.F., Capes, E.M., Diego-García, E., Zamudio, F.Z., Fuentes, O., Possani, L.D. and Valdivia, H.H. 2009. Characterization of hadrucalcin, a peptide from *Hadrurus gertschi* scorpion venom with pharmacological activity on ryanodine receptors. *British Journal of Pharmacology* 157:392-403.

Tang, X., Zhang, Y., Hu, W., Xu, D., Tao, H., Yang, X., Li, Y., Jiang, L. and Liang, S. 2010. Molecular diversification of peptide toxins from the tarantula *Haploplema hainanu* (*Ornithoctonus hainana*) venom based on transcriptomic peptidomic, and genomic analyses. *Journal of Proteome Research* 9:2550-2564.

Valdez-Velázquez, L.L., Quintero-Hernández, V., Romero-Gutiérrez, M.T., Coronas, F.I. and Possani, L.D. 2013. Mass fingerprinting of the venom and transcriptome of venom gland of scorpion *Centruroides tecomanus*. *PLoS ONE* 8:e66486.

Vargas-Jaimes, L., Xiao, L., Zhang, J., Possani, L.D., Valdivia, H.H. and Quintero-Hernández, V. 2017. Recombinant expression of intrepicalcin from the scorpion *Vaejovis intrepidus* and its effect on skeletal ryanodine receptors. *Biochimica et Biophysica Acta – General Subjects* 1861:936-946.

Zamudio, F.Z, Gurrola, G.B., Arevalo, C., Sreekumar, R., Walker, J.W., Valdivia, H.H. and Possani, L.D. 1997 Primary structure and synthesis of Imperatoxin A (IpTx(a)), a peptide activator of Ca2+ release channels/ryanodine receptors. *FEBS Letters* 405:385-389.

Zhang, L., Shi, W., Zeng, X.C., Ge, F., Yang, M., Nie, Y., Bao, A. & Wu, S.E.G. 2015. Unique diversity of the venom peptides from the scorpion *Androctonus bicolor* revealed by transcriptomic and proteomic analysis. *Journal of Proteomics* 128:231–250.

Zhong, J., Zeng, X.C., Zeng, X., Nie, Y., Zhang, L., Wu, S. and Bao, A. 2017. Transcriptomic analysis of the venom glands from the scorpion *Hadogenes troglodytes* revealed unique and extremely high diversity of the venom peptides. *Journal of Proteomics* 150:40-62.

Zhu, S., Darbon, H., Dyason K., Verdonck, F. and Tytgat, J. 2003 Evolutionary origin of the inhibitor cystine knot peptides. *FASEB Journal* 17:1765-1767.

Zobel-Thropp, P.A., Correa, S.M., Garb, J.E. and Binford, G.J. 2014 Spit and venom from scytodes spiders: a diverse and distinct cocktail. *Journal of Proteome Research* 13:817-835.
